# Links Between Inflammatory Bowel Disease and Chronic Obstructive Pulmonary Disease

**DOI:** 10.3389/fimmu.2020.02144

**Published:** 2020-09-11

**Authors:** April L. Raftery, Evelyn Tsantikos, Nicola L. Harris, Margaret L. Hibbs

**Affiliations:** Department of Immunology and Pathology, Central Clinical School, Monash University, Melbourne, VIC, Australia

**Keywords:** inflammatory bowel disease, Crohn's disease metabolites, chronic obstructive pulmonary disease, microbial dysbiosis, gut-lung axis

## Abstract

Inflammatory bowel disease (IBD) and chronic obstructive pulmonary disease (COPD) are chronic inflammatory diseases of the gastrointestinal and respiratory tracts, respectively. These mucosal tissues bear commonalities in embryology, structure and physiology. Inherent similarities in immune responses at the two sites, as well as overlapping environmental risk factors, help to explain the increase in prevalence of IBD amongst COPD patients. Over the past decade, a tremendous amount of research has been conducted to define the microbiological makeup of the intestine, known as the intestinal microbiota, and determine its contribution to health and disease. Intestinal microbial dysbiosis is now known to be associated with IBD where it impacts upon intestinal epithelial barrier integrity and leads to augmented immune responses and the perpetuation of chronic inflammation. While much less is known about the lung microbiota, like the intestine, it has its own distinct, diverse microflora, with dysbiosis being reported in respiratory disease settings such as COPD. Recent research has begun to delineate the interaction or crosstalk between the lung and the intestine and how this may influence, or be influenced by, the microbiota. It is now known that microbial products and metabolites can be transferred from the intestine to the lung via the bloodstream, providing a mechanism for communication. While recent studies indicate that intestinal microbiota can influence respiratory health, intestinal dysbiosis in COPD has not yet been described although it is anticipated since factors that lead to dysbiosis are similarly associated with COPD. This review will focus on the gut-lung axis in the context of IBD and COPD, highlighting the role of environmental and genetic factors and the impact of microbial dysbiosis on chronic inflammation in the intestinal tract and lung.

## Introduction

Inflammatory bowel disease (IBD) and chronic obstructive pulmonary disease (COPD) are chronic inflammatory diseases that affect the gastrointestinal tract and respiratory system, respectively, with both being characterized by recurrent disease cycles that result in tissue damage and worsening of disease symptoms. As mucosal epithelial sites, the gastrointestinal and respiratory tracts share structural similarities which may result in part from common embryonic origin in the primitive foregut (1). Its hypothesized that these structural similarities may account for inherent parallels in the immune responses at these two sites and contribute to the dynamic involvement of the gut-lung axis in inflammation.

## Chronic Inflammatory Diseases

### Inflammatory Bowel Disease

**Figure 1:**
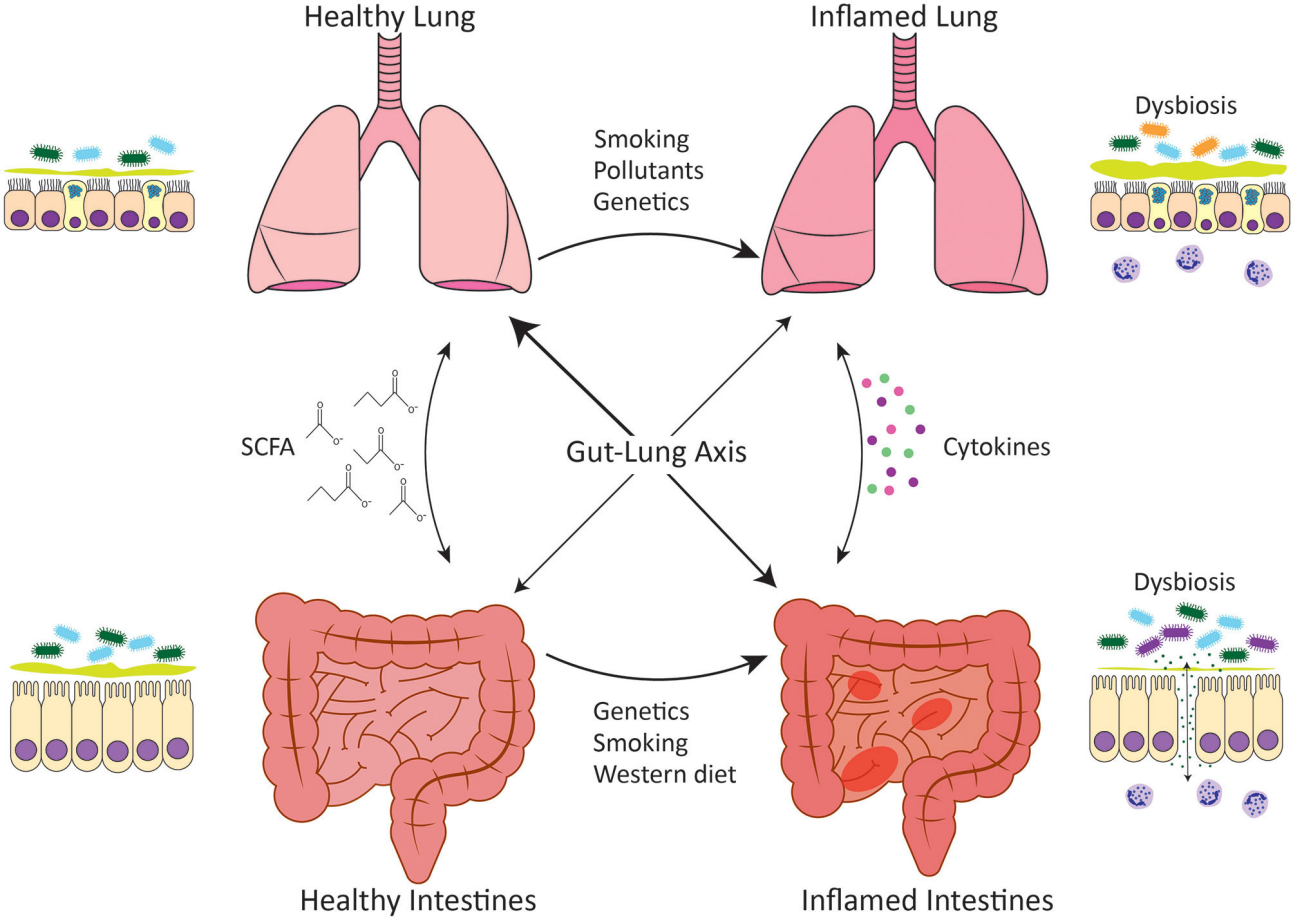
Gut-lung axis. Communication between the intestines and the lungs occurs in both healthy situations and disease settings. In healthy individuals, both the intestines and the lungs harbor diverse microbial communities that have evolved to complement the host and predominately comprise bacteria of the Bacteroidetes and Firmicutes phyla. The gut microbiota performs key functions such as the generation of SCFA from the host's diet, which play an important role in homeostatic maintenance. Microbial dysbiosis occurs in association with chronic inflammatory diseases such as IBD and COPD and leads to loss of epithelial barrier integrity and inappropriate immune responses directed against the microbiota. Dysbiosis is characterized by reduced diversity of *Firmicutes* spp. in IBD and the expansion of *Proteobacteria* spp. in COPD. Genetic variations as well as environmental stimuli such as cigarette smoke or a Western diet have been linked to intestinal and lung microbial dysbiosis. A healthy, fiber-rich diet promotes intestinal, and respiratory health.

IBD is an umbrella term that describes chronic relapsing inflammation of the gastrointestinal tract; the major types being Crohn's disease metabolites (CDM) and ulcerative colitis (UC). CD is characterized by transmural, non-continuous, and non-caseating granulomatous inflammation that can occur at any point along the entirety of the gastrointestinal tract, however, inflammation most commonly manifests in the terminal ileum (2, 3). UC is characterized by continuous inflammation that originates in the rectum and progresses proximally. Unlike CD, the inflammation in UC only affects the mucosa and submucosa and solely manifests in the colon (4, 5). The etiology of IBD has not been fully elucidated, however, a complex interplay of genetic susceptibility, environmental risk factors, inappropriate immune responses directed against the microbiota, intestinal barrier hyperpermeability, and dysbiosis of commensal microbiota of the intestines are thought to contribute to pathogenesis (6) (Figure 1).

Presently there are no curative treatments for IBD. With current management strategies, 10–35% of CD patients will require surgery within the first year of diagnosis, and up to 60% will require surgery within a decade of initial diagnosis (7). For UC, 30% of patients will require a colectomy within 10 years of diagnosis (8). Further research into the mechanisms driving IBD is needed to identify novel therapeutic targets, and a fuller understanding of the role of intestinal microbiota in IBD could provide some valuable insights in pursuit of this.

### Chronic Obstructive Pulmonary Disease

COPD is a progressive and largely irreversible disease that is characterized by prolonged inflammation, tissue destruction, and airflow obstruction leading to the reduced functional capacity of the lungs. The disease is driven by chronic exaggerated inflammatory responses in the airways and parenchyma of the lungs in response to a noxious insult such as cigarette smoke or environmental pollutants or genetic factors such as alpha-1 antitrypsin deficiency (Figure 1). Airway inflammation drives airway remodeling leading to mucus metaplasia and airway obstruction as well as tissue destruction that results in the enlargement of the alveoli, also known as emphysema. Smoking is a major risk factor for the development of COPD, however, other factors such as chronic asthma, low birth weight, childhood respiratory tract infections, pulmonary tuberculosis, and occupational exposures to dusts have also been associated with COPD (9). The prevalence of COPD has been reported to be as high as 20% in never-smokers suggesting that other risk factors for COPD have been overlooked due to the focus on cigarette smoke (10). COPD is one of the leading causes of mortality worldwide accounting for 3 million deaths annually (11), and much like IBD, there is no curative treatment beyond lung transplantation although this is still up for debate (12).

### Linking IBD and COPD

Population based studies have identified an increased prevalence of IBD in patients with COPD, and an increased risk of mortality in patients with both COPD and CD (13–15). Furthermore, the risk of COPD patients developing either CD or UC is increased by 2.72 and 1.83, respectively, compared to healthy controls (13), results that have been confirmed in additional populations (16). Reciprocally, a retrospective study found that CD patients were at increased risk of dying from COPD (17). Additionally, a study in Quebec residents found that asthmatic and COPD patients had increased incidences of CD of 27 and 55%, respectively, compared to the general population in that province (18). There is also evidence that up to 60% of IBD patients have some degree of subclinical lung disease (19). COPD and CD share environmental risks, with cigarette smoke exposure being a major risk factor for both (20). However, this does not explain the increased prevalence of UC amongst COPD patients as cigarette smoke has been proposed to be protective against the development of UC in the general population (21). CD patients without chronic lung disease have reduced pulmonary function that correlates with the presence of sputum lymphocytosis and eosinophilia and inversely with CD activity index (22). This suggests that shared environmental risk factors alone are not sufficient to induce these comorbidities and that alternative mechanisms that link the intestine and lung are responsible.

The epithelium of the respiratory and gastrointestinal systems are derived embryonically from the primitive foregut (1). This shared origin likely underlies the ability of these two mucosal surfaces to act similarly as selective barriers, allowing for the translocation of gases or nutrients, whilst maintaining mutualistic relationships with the microbiota and keeping pathogens at bay. These similarities in function suggest that these tissues may also respond to disease-causing stimuli in the same way, which could account for the increased risk of CD development in COPD patients. Indeed, parallels exist in the immune systems at both mucosal sites and this is mirrored in the immunopathology of IBD and COPD. Both disease settings are characterized by increases in myeloid cells such as neutrophils, eosinophils and macrophages, as well as innate lymphoid cells (ILCs) and unconventional T cells such as γδ T cells, all of which are important in microbial interactions and maintenance of epithelial barriers. While this is beyond the scope of this review, the reader is directed to the following reviews for more information on the pathophysiology and immunopathology of these diseases (23–26). It is well-known that the intestinal epithelium is readily damaged in IBD (27), and increased intestinal permeability has now also been reported in COPD (28, 29). Whilst the association between IBD and COPD has been largely investigated at an epidemiological level, further research is required to elucidate the inflammatory mechanisms that link the intestine and the lung. This review will focus on the mechanisms linking IBD and COPD that pertain to mucosal microbial communities and dysbiosis in disease settings (Figure 1).

## Mucosal Microbial Communities and Dysbiosis in Disease Settings

### Healthy Intestinal Microbiota

The gastrointestinal tract is one of the largest surfaces of the body that is constantly in contact with environmental factors. At mucosal sites, such as the intestine, the immune system is primed by interactions with the microbiota and environmental antigens, and a fine homeostatic balance needs to be maintained to ensure quiescence toward harmless microbes whilst being able to promote a proinflammatory response against invading pathogens. The intestine is the most densely colonized surface of the human body with Bacteroidetes and Firmicutes representing the two most abundant bacterial phyla making up approximately 90% of all microorganisms of the gastrointestinal tract (30, 31). A mutualistic relationship exists between the intestinal microbiota and the human host. The intestine provides a nutrient-rich niche for the microbiota to inhabit, whilst the host benefits from the increased digestive capacity that the microbiota provides, from its ability to prime the immune system, and from a reduction in the available niche for potentially pathogenic micro-organisms (32). In addition, the intestinal microbiome contains more than 3.3 million non-redundant genes, 150-fold greater than the human gene complement, and these provide both metabolic and health benefits to the host (33).

The microbiota is not stagnant, and its composition can be altered by a variety of factors including diet, infection, inflammation, or antibiotics; and this shift in microbial communities is referred to as dysbiosis. Dysbiosis is often associated with IBD (34, 35) but is also observed in a variety of chronic inflammatory and autoimmune diseases including rheumatoid arthritis, psoriasis, neurodegenerative diseases, diabetes, allergic diseases, and asthma (36–42). At present, essentially nothing is known about the gut microbiota in COPD.

### Healthy Lung Microbiota

Historically, due to a reliance on culture-dependent techniques, the lungs were thought to be sterile. With the emergence of culture-independent techniques utilizing genetic sequencing, pulmonary microbial communities have been found in the healthy lung and characterization of the lung microbiota in the context of respiratory diseases has become possible (43). However, progress in our understanding of the lung microbiota is still limited by factors such as low microbial loads in the lower airways, contamination of samples from the upper airways and oral cavity, and sampling methods that over-represent the upper airways over the lower airways. This is important since the upper and lower airways harbor distinct microbial communities (44). A study comparing the microbial communities of both the oral cavity and the lower airways in healthy non-smokers and smokers identified bacteria that comprised the healthy lung microbiota and showed that lung bacterial communities were not significantly altered by smoking, although differences were apparent in the oral microbiota. Similar to the intestines, the most abundant bacterial phyla present in the lungs were Bacteroidetes and Firmicutes and they did not derive entirely from the mouth as the microbiota of the oral cavity was dominated by Firmicutes, Proteobacteria, and Bacteroidetes (44).

### Dysbiosis of Intestinal Microbiota in IBD

Dysbiosis has been associated with the development of IBD, although whether this is cause or effect is yet to be elucidated since most research to date has been correlative. Cross-sectional studies are the most common for assessing the microbiota of IBD patients but these only provide a snapshot in time (45). Instituting longitudinal studies could assist in establishing when dysbiosis occurs relative to the onset of intestinal inflammation and how this may change with the course of disease but this would require early and frequent sampling. Furthermore, the microbiome tends to be sequenced from fecal samples and while this is non-invasive for the IBD patient, this method could provide inaccurate measures, especially for CD patients where inflammation is often localized to the ileum or other portions of the gastrointestinal tract. This sampling method can also overlook mucosa-associated microbiota, and due to the variations in microbial composition that are apparent across the gastrointestinal tract, sampling methods need to be carefully considered when formulating studies (46). Nonetheless, the research that has been conducted to date has provided a solid foundation upon which future studies can expand to improve our understanding of dysbiosis in IBD.

Broadly, dysbiosis in IBD patients is associated with shifts promoting an increase in potentially proinflammatory bacteria and a decrease in protective bacteria. In CD patients, intestinal dysbiosis has been characterized by a reduction in diversity of species belonging to the Firmicutes phyla, a change that has been suggested to occur prior to disease onset (34, 47, 48). Furthermore, a specific reduction in relative abundance in *Dialister invisus* and *Faecalibacterium prausnitzii* species of the Firmicutes phylum and *Bifidobacterium adolescentis* of the Actinobacteria phylum, together with an increase in the mucolytic species *Ruminococcus gnavus* and *Ruminococcus torques* of the Firmicutes phylum is observed in CD patients compared to healthy controls (34, 49). The importance of intestinal microbiota in development of IBD is supported by common experimental mouse models, such as nucleotide-binding oligomerization domain-containing protein 2 (NOD2)-deficiency and IL-10-deficiency which ordinarily develop ileitis when maintained under standard housing conditions but display reduced disease penetrance in specific pathogen-free environments (50–53).

Adherent-invasive *Escherichia coli* (AIEC) is an organism that is commonly associated with CD. This bacterium is able to take advantage of gaps in host defense such as impaired bacterial recognition and defective intracellular killing, allowing AIEC to expand (54). AIEC have been found to induce granuloma formation in the inflamed ilea (55), a hallmark pathological feature of CD. These granulomas consist of multinucleated giant cells and epithelioid cells, both phagocytic cells of the macrophage lineage that activate T cells in an antigen-specific manner, that are surrounded by a B cell corona (56). Furthermore, it has been found that AIEC are able to survive and replicate within macrophages without inducing cell death, a process that also promotes increased secretion of proinflammatory tumor necrosis factor-α (TNF-α) (57), which is involved in the pathogenesis of CD.

A study that examined dysbiosis in an inducible model of ileitis using *Toxoplasma gondii* together with high dose indomethacin, found that severe ileitis was associated with a shift in microbial communities from populations dominated by species belonging to the Firmicutes phyla to those largely represented by species belonging to the Proteobacteria phyla (58). This dysbiosis was accompanied by translocation of AIEC and was similar to that observed in CD patients. While these data suggest that inflammation is sufficient to induce dysbiosis, it is also clear that genetic susceptibility plays a role since *T. gondii* induced heightened dysbiosis and AIEC invasion in mice lacking the ileitis susceptibility gene *NOD2*, while disease was significantly muted in mice lacking the proinflammatory C-C chemokine receptor 2 (CCR2), which are a model of ileitis resistance (58). Furthermore, it has been demonstrated in an alternative genetic knock out model of ileitis susceptibility that dysbiosis precedes the onset of ileitis (59).

Similar to CD, dysbiosis in UC is characterized by a reduction in species belonging to Firmicutes and Bacteroidetes phyla, and a concomitant increase in species belonging to Proteobacteria and Actinobacteria phyla (60). However, at the species level, the dysbiotic signature of UC is distinct from CD. UC patients exhibit a reduction in relative abundance of *Roseburia hominis* and *Faecalibacterium prausnitzii* (35), both butyrate-producing bacteria of the Firmicutes phylum whose abundance is inversely correlated with disease severity (35). A similar mechanism involving the interaction of genetic susceptibility, inflammation and microbial dysbiosis contributes to UC.

### Genetics of IBD and Its Role in Intestinal Dysbiosis

To date, more than 150 susceptibility genes have been identified for IBD, most of which are involved in the detection and clearance of microbial compounds (61). Three common variants of NOD2 are associated with an increased risk of developing CD, with a 2–4-fold increase for heterozygous mutations and a 20–40-fold increase for homozygous mutations (62, 63). NOD2 variants are the most common mutations in CD patients of Caucasian descent, being identified in 30% of patients (64). Furthermore, certain variants in NOD2 have been associated with more severe disease phenotypes including early-onset disease, ileitis, and strictures caused by fibrosis (65–67). NOD2 is a cytoplasmic molecule that senses the pattern-associated molecular pattern muramyl dipeptide (MDP) of gram positive and negative bacteria, stimulating the immune response via activation of the transcription factor NF-κB or induction of apoptosis (68). The three common variants found in CD patients all affect NOD2 binding to MDP resulting in loss of function (62). This is thought to cause diminished secretion of antimicrobial peptides, which can lead to dysbiosis and promote a mucosal immune response (69). Functional loss of NOD2 in macrophages may also induce IL-12 and IL-1β expression which leads to the promotion of type 1 immune responses and inflammation (51). Interestingly, not everyone harboring homozygous or compound heterozygous NOD2 variants develop CD (70). Furthermore, NOD2-deficient mice do not spontaneously develop CD-like intestinal inflammation but require a second genetic mutation and specific microbiota (71). This suggests that additional factors, be those microbial, genetic or other environmental factors, are required to promote disease onset in susceptible individuals.

Autophagy-related 16-like protein 1 (ATG16L1) is crucial for normal autophagy function in cells, which is a standard cellular recycling process for protein and organelle turnover that is upregulated during nutrient deprivation or cellular stress signals such as microbial infection. It has been reported that 33.2% of Caucasian CD patients are homozygous for the CD-associated polymorphism rs2241880 (T300A) increasing their risk of developing CD by 2.38-fold (72, 73). This single nucleotide polymorphism is a missense mutation near a caspase cleavage site, making ATG16L1 more sensitive to degradation resulting in diminished autophagy, impaired ability of monocytes to clear invading pathogens and increased production of IL-1β in response to MDP (74). Normal ATG16L1 activity in intestinal epithelial cells (IEC) is important for maintaining the intestinal barrier. The rs2241880 variant in CD patients has been associated with Paneth cell abnormalities, specifically an impaired secretory granule pathway and increased production of proinflammatory mediators (75). Atg16l1-deficiency in IEC has been associated with increased susceptibility to colitis in mice, with increased CD4^+^ T cells and increased secretion of proinflammatory cytokines such as TNF-α, interferon-γ (IFN-γ) and IL-1β. Thus, diminished autophagy may induce susceptibility through alterations to both immune cell activity and intestinal barrier function.

Changes in *NOD2* and *ATG16L1*, as well as other genes involved in the intestinal epithelial barrier, microbial sensing, and antimicrobial activity in IBD, demonstrate the impact of genetics on the intestinal microbiota. Deficiencies in such genes are a mechanism by which dysbiosis can precede development of IBD and drive intestinal inflammation.

### Dysbiosis in COPD

#### Lung Dysbiosis is Observed in COPD

Chronic inflammatory lung diseases such as COPD and asthma have been associated with dysbiosis of the lung microbiota with the outgrowth of pathogenic bacteria. Mucus hypersecretion and lower respiratory tract infections in COPD have been associated with accelerated decline in lung function, indicating that the lung microbiota plays an important role in COPD pathogenesis (76, 77). The microbiota of the bronchial secretions from COPD patients predominately comprises members of the Proteobacteria, Firmicutes, and Actinobacteria phyla (78). Specifically, studies that have assessed the lung microbiota of COPD patients suggest that their bacterial communities differ from those of healthy individuals, with an expansion of *Hemophilus* spp. *Afipia, Brevundimonas, Curvibacter, Moraxella, Neisseria* and *Undibacterium* spp. of the Proteobacteria phylum, *Corynebacterium* spp. of the Actinobacteria phylum, *Capnocytophaga* spp. of the Bacteroidetes phylum, and *Leptolyngbya* spp. of the Cyanobacteria phylum, as well as a reduction in microbial community diversity compared to healthy individuals (79–82). Patients with more severe COPD have a less diverse lung microbiota but expansion of more pathogenic microbes (78). Furthermore, during acute exacerbations of COPD, the lung microbiota are more unstable; these exacerbations tend to be associated with reduced species diversity, an increased relative abundance of Proteobacteria mainly due to increased *Moraxella* spp. and a decreased relative abundance of species belonging to the Firmicutes phyla (82, 83). Changes in the core microbiota during acute exacerbations of COPD allow for an expansion of respiratory pathogens including *Acinetobacter* spp. and *Klebsiella* spp. highlighting the important role of the commensal lung microbiota in protecting against the colonization of pathogenic microbes (83). It is worth highlighting that the exact nature of dysbiosis within the lung microbiota that occurs during acute exacerbations of COPD is dependent on the cause of the exacerbation, with specific differences noted between bacterial and eosinophilic exacerbations (characterized by bacterial dysbiosis and elevated sputum eosinophils respectively). The characteristic decrease in species diversity and relative abundance of species belonging to the Firmicutes phyla, alongside an increase in species belonging to the Proteobacteria phyla is more pronounced in bacterial exacerbations (82). Bacterial exacerbations also have a significant decrease in *Streptococcus* spp. and an increase in *Hemophilus* spp. whilst eosinophilic exacerbations exhibit a decrease in the Proteobacteria:Firmicutes ratio (82). Changes in the composition of the lung microbiota are associated with changes in local inflammatory responses, the most significant being the negative correlation between species diversity and CXCL8, which indicates reduced species diversity is associated with an influx in neutrophils (82). Interestingly, in lung transplants, the microbiota influences the immune response with Firmicutes-dominated and Proteobacteria-dominated dysbiosis being proinflammatory, Bacteroidetes-dominated dysbiosis being associated with tissue remodeling, and a balanced microbial community being associated with homeostasis (84). Collectively, these studies show that respiratory microbial communities can regulate inflammation in the lungs.

#### Intestinal Dysbiosis Influences Lung Health

Recent research has shown that the intestinal microbiota is important in reducing the risk of lung inflammation by supporting mucosal immunity. Studies have shown that depletion of intestinal bacteria through antibiotic treatment, renders mice more susceptible to Pneumonia infection and respiratory inflammation (85, 86). Reciprocally, viral and bacterial respiratory infections are able to drive dysbiosis of the intestinal microbiota demonstrating that respiratory inflammation can influence the intestine (87, 88). In mice lacking an intestinal microbiota, their alveolar macrophages have an altered transcriptome which results in decreased phagocytic activity and bacterial killing (85, 86). Dysbiosis of the intestinal microbiota has been shown to influence the composition of the respiratory microbiota through changes in circulating inflammatory cytokines and translocation of intestinal microbiota to the airways Figure 1, although this has only been demonstrated in severe sepsis models and has not yet been established in COPD. It has been shown that segmented filamentous bacteria (SFB) in the intestines induced Th17 responses and IL-22 production in the lungs and protected against respiratory infection with *S. pneumoniae* with reduced bacterial burden and lung inflammation (89). In a murine model of sepsis, an increased abundance of gut-specific bacteria in the lungs has been observed, with there being greater similarities between the communities found in the intestines and lungs than in sham mice (90). A meta-analysis of sixteen studies found that infection with *H. pylori*, which colonizes the human gastric mucosa, was associated with an increased risk of COPD (91). During acute exacerbations of COPD there is a significant decrease in categories of bacteria as defined by operational taxonomic units (OTUs) in the intestinal microbiota (83). Beyond assessing *H. pylori* infection in COPD patients, and a recent study examining the intestinal microbiota in acute exacerbations of COPD in a small group of patients (83), the intestinal microbiota has not been investigated in COPD. This would be of particular interest since cigarette smoking, which is strongly associated with COPD, has been linked with dysbiosis of fecal microbiota in CD patients, characterized by an increase in the relative abundance of Bacteroides and Prevotella (92).

### Cigarette Smoke Exposure is a Risk Factor for Both COPD and CD

Cigarette smoke is the most important risk factor in COPD, with approximately 80% of COPD patients being past or current smokers. Smoking can have prolonged effects on lung inflammation which can persist years after smoking cessation, despite the slowed decline in lung function and better survival (93). In addition, active smoking is also associated with higher mortality rates in COPD patients (94). Both active and passive smoking is the most well recognized environmental risk factor for CD being associated with a 2-fold increased risk of disease (95). It is linked with early onset of disease, as well as more aggressive disease progression with an increase in the occurrence of strictures and fistulas and the increased likelihood of a need for surgical intervention (96–98). Smoking may also influence the locus of inflammation, increasing occurrence in the ileum as opposed to the colon (99). An intervention study investigated the effect of quitting smoking on CD severity and found that patients who stopped for at least a year were less likely to relapse (100). The association between smoking and CD highlights the potential crosstalk between the lungs and intestines, although, the possibility that noxious agents from cigarettes can reach the intestines via the oral route cannot be ignored as an additional CD risk mechanism (101).

Smoking also alters the composition of the intestinal microbiota not only in CD patients but also in smokers without IBD (102). Following smoking cessation, the fecal microbiota is altered in non-IBD individuals with an increased relative abundance of species belonging to Firmicutes and Actinobacteria phyla and a decreased relative abundance of species belonging to Bacteroidetes and Proteobacteria phyla (103). CD patients who smoke exhibit intestinal dysbiosis characterized by a higher Bacteroides:Prevotella ratio compared to non-smokers and healthy smokers (92). Animal studies that examined the impact of smoke exposure on the intestinal microbiota found that smoking increased the relative abundance of *Clostridium clostridiforme* with a decreased relative abundance of *Lactoccoci* spp. and *Ruminococcus albus* of the Firmicutes phylum and *Enterobacteriaceae* spp. in the cecum compared to control mice (3, 104). Collectively, these studies suggest that dysbiosis of the intestinal microbiota could be another mechanism by which cigarette smoke might increase the risk of CD development.

A study of chronic smoke exposure in mice compared the microbiota across the ileum, cecum, and distal colon finding changes in microbiota (105). The authors described increased activity of *Lachnospiraceae* spp. in the cecum and colon, which is of particular interest since it has been reported that *Lachnospiraceae* spp. can promote macrophage recruitment to the colon (106). This study also demonstrated that chronic smoke exposure may impact the intestinal microbiota by altering mucus profiles and the local immune environment. They found that cigarette smoke increased the secretion of the two major ileal mucins, Muc2 and Muc3, and enhanced the cell surface expression of the anti-adhesive Muc4 (105). However, at present it is unclear if these changes are a result of dysbiosis, or of cigarette smoke itself.

Cigarette smoke has been found to increase intestinal barrier permeability in the ileum, but not the colon (107). These changes are associated with intestinal villi atrophy, bacterial translocation and abnormal tight junction proteins, with evidence that they were mediated through NF-κB signaling (107, 108). Cigarette smoke has also been found to alter Paneth cell function in mice through reduced antimicrobial peptide expression and reduced bactericidal capacity, which leaves mice more susceptible to bacterial infection (108). Changes in the ileum, but not the colon, may explain why cigarette smoke increases the risk of developing CD whilst conceivably offering protection against UC (95). Certain susceptibility genes have been associated with epithelial barrier defects in CD patients who smoke. A promoter variant in the gene encoding the aryl hydrocarbon receptor (AHR) has been linked to increased risk of intestinal hyperpermeability, with cigarette smoking further increasing this risk (109). From an immunological perspective, cigarette smoke induces an IL-17 response with increases in Th17 cells and neutrophils in the lungs and circulation (110). This enhances intestinal Th17 cells and neutrophils, as well as IL-17-producing type 3 innate lymphoid cells (ILC3s), in a manner that is dependent on neutrophil recruitment via IL-17A (110). Thus, in addition to changes in the intestinal epithelial barrier, cigarette smoke can promote intestinal inflammation which is already augmented in individuals who are genetically susceptible to IBD.

### Non-bacterial Microbiota in IBD and COPD

To date most studies examining the microbiota in IBD and COPD have focused on bacteria, however, the microbiota also encompasses fungi and viruses. Whilst this review primarily discusses the role of bacterial dysbiosis in IBD and COPD these other microorganisms cannot be ignored. There is evidence to suggest that there are shifts in the intestinal viral and fungal communities during IBD and alterations to pulmonary communities in COPD. However, considerably more research is needed to determine the role of viruses and fungi in the gut-lung axis especially in the context of IBD and COPD.

#### The Virome and Mycobiome May Contribute to IBD

The intestinal virome is predominately comprised of bacteriophages (111), therefore the interactions between viruses and bacteria during IBD could play a role in dysbiosis and disease pathogenesis (112). The most abundant bacteriophages of the intestine include the Caudovirales order and Microviridae family (113–115) and perhaps not surprisingly, viral dysbiosis in IBD patients is characterized by an increase in Caudovirales species (115, 116). This expansion is associated with reduced bacterial diversity and does not appear to occur secondary to changes in bacterial populations suggesting that the virome may contribute to bacterial dysbiosis and inflammation in IBD (115). However, virome research in IBD is in its infancy and more studies are required to elucidate how changes in intestinal viruses may impact upon other intestinal microorganisms and intestinal inflammation.

A potential role for fungi in IBD pathogenesis was first proposed in 1988 when antibodies directed against Saccharomyces cerevisiae were identified in the blood of CD patients (117). Furthermore, several IBD susceptibility genes are involved in anti-fungal immune responses such as CARD9 and CLEC7A (61). Fungal dysbiosis in IBD has been characterized by an increased Basidiomycota:Ascomycota ratio, decreased proportion of Saccharomyces cerevisiae and an increased proportion of Candida albicans (118). Similar to the virome, further research is needed to understand how changes in the mycobiome during IBD may impact upon other microorganisms and inflammation.

#### The Virome and Mycobiome in COPD

Similar to the gastrointestinal tract the respiratory tract consists of bacteriophages and eukaryotic viruses (119, 120). COPD patients have a heightened viral load in their lungs with an increased abundance of influenza, cytomegalovirus, and Epstein-Barr virus, the latter of which has been associated with pulmonary fibrosis, a feature of COPD (121–123). More non-targeted approaches are required to define other viruses that may be involved in COPD pathogenesis and dysbiosis.

The most abundant fungi in healthy lungs are of the Davidellaceae family and the genera Cladosporium, Eurotium, Penicillium, and Aspergillus, although many other species including Candida spp. are present as well (124). Compared to healthy individuals, COPD patients have an increased relative abundance of Candida spp. in their lungs (125). Furthermore, the enhanced abundance of Aspergillus, Candida, Phialosimplex, Penicillium, Cladosporium, and Eutypella has been associated with severe exacerbations of COPD (126). Of the chronic pulmonary diseases, COPD is one of the least studied in the context of the mycobiome and more research is required to understand how the mycobiome is altered in COPD patients. Furthermore, the relationship between the non-bacterial microbes of the lung and gut and their role in the gut-lung axis have been poorly considered and are an area for future research.

## Factors Linking IBD and COPD

### Dietary-Derived Metabolites Are Protective in IBD and COPD

Certain macro- and micro-nutrients have been inversely associated with the development of CD (127), with dietary fiber being the most extensively researched. Soluble-fiber from fruits and vegetables as opposed to insoluble fiber from whole grains and cereals has shown protection against CD (128). Non-digestible carbohydrates are fermented by saccharolytic bacteria in the gastrointestinal tract into metabolites known as short-chain fatty acids (SCFA), which include acetate, propionate, and butyrate. Acetate and propionate are produced by Bacteroidetes and butyrate by Firmicutes and these SCFA can be immunomodulatory by preventing the transcription of proinflammatory mediators. Butyrate in particular is an energy source for IEC thereby promoting intestinal barrier integrity (129), and thus it is not surprising that a decrease in butyrate-producing bacteria is a characteristic of intestinal dysbiosis in IBD (35). Experimental models of IBD have shown that dietary SCFA reduce inflammation, specifically via decreases in proinflammatory mediators such as TNF-α and nitric oxide synthase which correlates with increased concentrations of butyrate and propionate in the luminal contents of the intestines (130, 131). However, the efficacy of SCFA in IBD patients has been brought into question due to the reduced responsiveness of their peripheral blood mononuclear cells to *n*-butyrate following toll-like receptor-2 (TLR-2) activation (132). Furthermore, for UC patients, butyrate enemas have shown no clinical benefit (133). This could be due to the finding that monocarboxylate transporter 1, which is responsible for the uptake of butyrate in the intestine, is downregulated in response to proinflammatory cytokines and its expression is reduced in the inflamed mucosa of IBD patients and in a rat model of colitis (134). Interestingly, studies examining the fecal contents of CD patients found that disease activity or localization was not affected by SCFA concentration, although CD disease activity was inversely correlated to levels of the medium-chain fatty acid hexanoate (135, 136). Contradictory to these studies, a clinical trial examining the effects of soluble fiber supplements showed that high fiber could reduce disease activity index in CD patients (137). Discrepancies in research may reflect the heterogeneity of CD as well as differences in diets, and thus, further research is required to elucidate the effects of the different components of diet on intestinal inflammation.

It is now known that SCFA can have immunomodulatory effects beyond the intestines where they are produced, promoting anti-inflammatory responses elsewhere in the body. Dysregulated SCFA production and absorption has been implicated in a variety of neurological, metabolic, allergic, and autoimmune diseases (138–141). In the context of the lungs, increased dietary fiber intake is associated with improved lung function in the general population and a reduced risk of developing COPD (142, 143). Increased intake of vegetables, which are high in soluble fiber, is associated with improved COPD symptoms such as breathlessness, as well as reduced risk of developing COPD (144). Similar to vegetables, high dietary fruit intake has also been associated with improved COPD outcomes and reduced incidence of COPD (145–148). Despite this correlation between increased dietary fiber intake and protection against COPD, there have been few reports on the efficacy of SCFA specifically in COPD pathogenesis highlighting that this is an understudied area worthy of further research. High fiber diet-producing SCFA have been shown to be immunomodulatory in asthma responses by enhancing the production of dendritic cells that seed the lungs but have an impaired ability to promote pathogenic type 2 immune responses (149). A more recent study building on these findings has shown that high fiber diets protect against influenza by enhancing the generation of Ly6c^−^ patrolling monocytes from progenitors. This led to an increase in alternatively activated macrophages in lungs and restrained neutrophil recruitment while simultaneously enhancing influenza-specific CD8 T cells responses (150). Similar mechanisms may be associated with the protection created by high fiber diets in COPD where neutrophils play a key pathogenic role in the inflamed lungs. COPD patients exhibit poor responses to influenza vaccination (151, 152) and thus, diet modulation may be a mechanism to improve vaccination outcomes for this susceptible population. In all, modulation of intestinal microbiota with high fiber diets might be beneficial to IBD and COPD patients (Figure 1), however, further research is required to determine how efficacious this strategy would be.

### Dietary Fat Can Alter the Gut Microbiota and Influence Disease

Foods high in saturated fat or “Western” diets have been associated with a variety of autoimmune and chronic inflammatory disease including IBD and COPD (142, 153, 154). A “Western” diet can influence the composition of the intestinal microbiota, promote intestinal barrier permeability, and enhance inflammation (155–157). Generally, fat intake is able to induce proinflammatory responses through the increase in cytokines, including TNF-α and IL-6, and neutrophils in circulation, all of which play a pathogenic role in IBD and COPD (155, 158). Intestinal dysbiosis induced by fat intake is characterized by an increased Firmicutes:Bacteroidetes ratio and the promotion of endotoxemia, which induces intestinal barrier hyperpermeability (156, 157, 159). In CD patients, the changes induced by a high fat diet that contribute to dysbiosis include increased intestinal barrier permeability, reduced mucus layer thickness and increased NOD2, TLR5, and carcinoembryonic antigen-related cell adhesion molecule 6 (CEABAC6) expression, all of which allow for AIEC colonization (49, 160). Generally, a high fat diet tends to be associated with a low fiber diet, and thus, a “Western” diet may contribute to IBD and COPD pathogenesis not only through direct proinflammatory mechanisms but also indirectly through a reduction in the anti-inflammatory benefits of SCFA Figure 1.

### Vitamin D Alters the Microbiota and May Have Therapeutic Benefits in CD and COPD

Vitamin D deficiency commonly occurs in IBD patients and has been associated with diagnosis and the need for surgical intervention (161). In keeping with this, colitis-prone IL-10-deficient mice exhibit a decline in vitamin D receptor (VDR) expression that correlates with colitis symptoms (162). In addition, mouse models unable to produce the active form of vitamin D, 1,25-dihydroxycholecalciferol [1,25(OH)_2_D_3_], or lacking the VDR are more susceptible to DSS-induced colitis and this is associated with intestinal dysbiosis characterized by an increase in species of the Proteobacteria phylum and a decrease in species of the Firmicutes phylum (163, 164). VDR signaling regulates numerous antimicrobial processes including the expression of β-defensins, cathelicidin antimicrobial peptides, and ATG16L1 (162). In IBD patients, reduced ATG16L1 expression due to deficiency in VDR signaling promotes an overrepresentation of intestinal *Bacteroides* and a decrease in butyrate-producing bacteria (162). Interestingly, treating human IEC with butyrate upregulates VDR expression, a phenomenon that also translated to IL-10-deficient mice that were given butyrate, suggesting a close link between the microbiota and vitamin D signaling (162). Vitamin D supplementation in CD patients results in an increase in Firmicutes species correcting some of the dysbiosis that occurs in CD (165). In COPD patients, increased vitamin D intake is positively associated with improved lung function, and like IBD, vitamin D deficiency is associated with COPD (166, 167). This could relate to the effect of vitamin D on the intestinal microbiota. Additionally, vitamin D plays a role in macrophage activation and shaping the lung microbiota promoting reduced bacterial richness (168, 169). VDR-deficient mice exhibit increased inflammation in the lungs with up-regulation of matrix metalloproteinase-2 (MMP-2), MMP-9, and MMP-12, the development of emphysema and a decline in lung function mimicking COPD in humans (170). Studies where mice are fed vitamin D have shown that vitamin D reduces the abundance of respiratory pathobionts, such as *Pseudomonas*, and increases the secretion of murine β-defensin-2 in the lungs (171). To date, very little has been done examining the impact of vitamin D supplementation on the lung microbiota. One study in cystic fibrosis patients found that the sputum of vitamin D insufficient patients was enriched for *Bacteroides* and there was a significant difference between the lung microbiota of these patients and those who were vitamin D sufficient (172). It is clear that the immunomodulatory and microbiota-regulating effects of vitamin D can strongly influence inflammation in both the intestine and the lungs. Given that vitamin D deficiency is associated with both IBD and COPD, vitamin D supplementation should be trialed more extensively in these patient cohorts, specifically in patients with comorbid IBD and COPD.

## Therapeutic Targeting of the Intestinal Microbiota

### Antibiotics for IBD: the Yin and Yang

Early-life antibiotic treatment has been associated with early onset CD via enhanced pathogenicity of helper T cells (173). This link is particularly strong in children who have received multiple doses of antibiotics or antibiotics during their first year of life (174, 175). Antibiotics cause dysbiosis of intestinal microbiota and a reduction in bacterial diversity, and this may be a potential mechanism by which antibiotic therapy in early life could result in the development of CD in genetically susceptible individuals (176). Furthermore, short-term antibiotic treatment may have prolonged effects, up to at least 2 years post-therapy (177). In infancy, the intestinal microbiota between individuals can be highly variable, before converging to more similar phyla in adulthood (178). This may in part explain the profound impact of antibiotic treatment on the microbiota in early childhood.

While antibiotics given in early life may promote CD, they have been used to treat CD with varying levels of success, and this appears to be dependent on disease location and severity as well as the type of antibiotic (179). Differences in populations of commensal bacteria between the ileum and colon most likely contribute to the lack of response of ileitis patients to a variety of antibiotics (179, 180). Patients who had undergone ileal resection and were treated with metronidazole, an antibiotic that is usually ineffective in patients with ileitis (180), exhibited a delay in symptomatic recurrence (181). A systematic review of antibiotic therapy in CD patients found that antibiotics likely have a modest effect that may not be clinically relevant (182). Furthermore, to maintain antibiotic treatment efficacy and prevent relapse, long-term treatment is required, as with all therapies for CD (183, 184). All in all, antibiotics in early life may increase the risk of CD development in susceptible individuals but may be beneficial as a therapeutic in established disease.

Unlike CD, antibiotic exposure is not associated with an increased risk of developing UC (173) and indeed antibiotics are effective as an adjunctive to conventional therapies, including corticosteroids and 5-aminosalycilic acid (185). This emphasizes the pathogenic role that bacterial dysbiosis plays in UC and suggests that more therapeutics targeting both inflammation and dysbiosis could benefit a large proportion of UC patients.

### Antibiotics for COPD Are Used to Manage Disease Exacerbations

Persistent and recurrent infections contribute to the progression of COPD through the induction of further chronic inflammation. Antibiotics are commonly used to treat acute exacerbations of COPD, however, the efficacy of antibiotic treatment in mild to moderate exacerbations is still in debate (186, 187). Treatment of COPD patients with antibiotics enhances respiratory microbiota diversity, decreases the relative abundance of Proteobacteria species and increases the relative abundance of Firmicutes species. These changes somewhat correct the dysbiosis associated with COPD, an effect that is maintained post-therapy (80, 82). Contrary to antibiotic therapy, corticosteroids are associated with a decrease in species diversity and an increase of Proteobacteria over Firmicutes corresponding to an increase in *Hemophilus* spp. and *Moraxella* spp. and a decrease in *Streptococcus* spp. changes that are associated with COPD pathogenesis (82). The differing effects of antibiotics and corticosteroids on the lung microbiota suggest that antibiotics are able to partially restore lung microbial communities whilst corticosteroids may further promote dysbiosis. Macrolides are the most commonly prescribed antibiotics for COPD due to both anti-inflammatory and immunomodulatory effects, however, the mechanisms by which macrolides exert these effects have not been elucidated (188). Relatively few COPD patients are treated with antibiotics long-term (189), however, the few studies that have been conducted suggest that prophylactic antibiotic treatment can reduce exacerbations in COPD patients (190). Nonetheless, further studies are required to understand whether long-term antibiotics are efficacious and the impact they have on the lung and intestinal microbiota.

### Fecal Microbiota Transplants, a Possible Treatment Strategy

Fecal microbial transplants are effective in the treatment of *Clostridium difficile* infection, an intestinal disease that is linked to intestinal bacterial dysbiosis (191). Success is associated with an expansion in bacterial diversity including increases in Bacteroidetes, Firmicutes and other butyrate-producing bacteria and a decrease in Proteobacteria (192). In patients with UC, fecal microbial transplants have some initial benefit by promoting a change in colonic microbiota at the phylum level with a decrease in the relative abundance of Proteobacteria species and an increase in Bacteroidetes species. While this partially corrects the dysbiotic changes that are associated with UC, these changes were not prolonged and did not translate to a vast clinical improvement (193). Another study where patients were treated with antibiotics prior to fecal microbial transplant had better clinical outcomes resulting in remission (194). Patients who respond to fecal microbial transplants have been characterized by distinct microbial profiles compared to non-responders. Alterations in microbiota in responders is characterized by an increase in bacterial diversity as well as a shift in composition toward that of the donor feces (195, 196). Lack of response is associated with the presence of *Fusobacterium* spp. and *Sutterella* spp. suggesting that patients should be screened prior to treatment (196). Studies examining the efficacy of fecal microbial transplants for UC have yielded variable results, which could be due to a lack of consistency in methodology as well as the heterogeneous nature of UC and more work is required to establish if this could be an efficacious treatment strategy.

In the context of CD, there has been far less research into the efficacy of fecal microbial transplants and at present it is not clear if this could be a management strategy for CD patients (197, 198). With respect to COPD, no fecal or respiratory microbiota transplant studies have been conducted and thus it is not yet known if these strategies could be a viable option for this disease.

## Conclusion

Microbial dysbiosis has a pivotal role in the development of IBD and COPD impacting on the intestinal and respiratory epithelial barriers and promoting damaging immune responses. Circulating microbial products and their metabolites are altered during dysbiosis and likely represent a significant component of the gut-lung axis. Shifts in these factors, where they may be produced at one site and act at another, provides a mechanism for organ crosstalk in disease and the comorbid presentation of IBD and COPD. Currently there are no curative treatments for either disease. However, elucidating the mechanisms by which the intestinal and respiratory microbiota drive inflammation and promote changes in mucosal epithelial barriers could provide new insights into disease pathogenesis and help to improve current treatment strategies and identify novel therapeutic targets. These may include approaches that target the microbiota such as diet, antibiotics or fecal microbiota transplants which may be able to modulate inflammation in the intestines and lungs. Most microbiota studies have focused on either IBD or COPD and have largely ignored patients with both diseases. Additionally, there are no reports of gut microbiota alterations in COPD, with published studies focused solely on the lung metagenome. Future studies into patients that harbor COPD, as well as patients with both mucosal inflammatory diseases will provide a more complete understanding of the microbiota in the gut-lung axis in health and disease. Furthermore, increasing awareness and understanding of the links between IBD and COPD will improve clinical management and more timely detection of comorbid disease in affected patients.

## Author Contributions

AR, NH, and MH conceived and planned the review. AR wrote the first draft of the manuscript. ET, NH, and MH provided editorial comment. All authors contributed to the article and approved the submitted version.

## Conflict of Interest

The authors declare that the research was conducted in the absence of any commercial or financial relationships that could be construed as a potential conflict of interest.
